# Supplementary material for: Generation, localization and functions of macrophages during the development of testis
Source: Nat Commun. 2020 Sep 1;11:4375. doi: 10.1038/s41467-020-18206-0 (PMC7463013; doi:10.1038/s41467-020-18206-0)
Supplement: Supplementary file 1 — Supplementary Information [file 41467_2020_18206_MOESM1_ESM.pdf]

Supplementary Information

Generation, localization and functions of macrophages during the development of testis

Emmi Lokka, Laura Lintukorpi, Sheyla Cisneros-Montalvo, Juho-Antti Mäkelä, Sofia Tyystjärvi, Venla Ojasalo, Heidi Gerke, Jorma Toppari, Pia Rantakari*, Marko Salmi*

*Correspondence: marko.salmi@utu.fi, pia.rantakari@utu.fi

Table of Contents:

Supplementary Figures 1-12

Supplementary Table 1

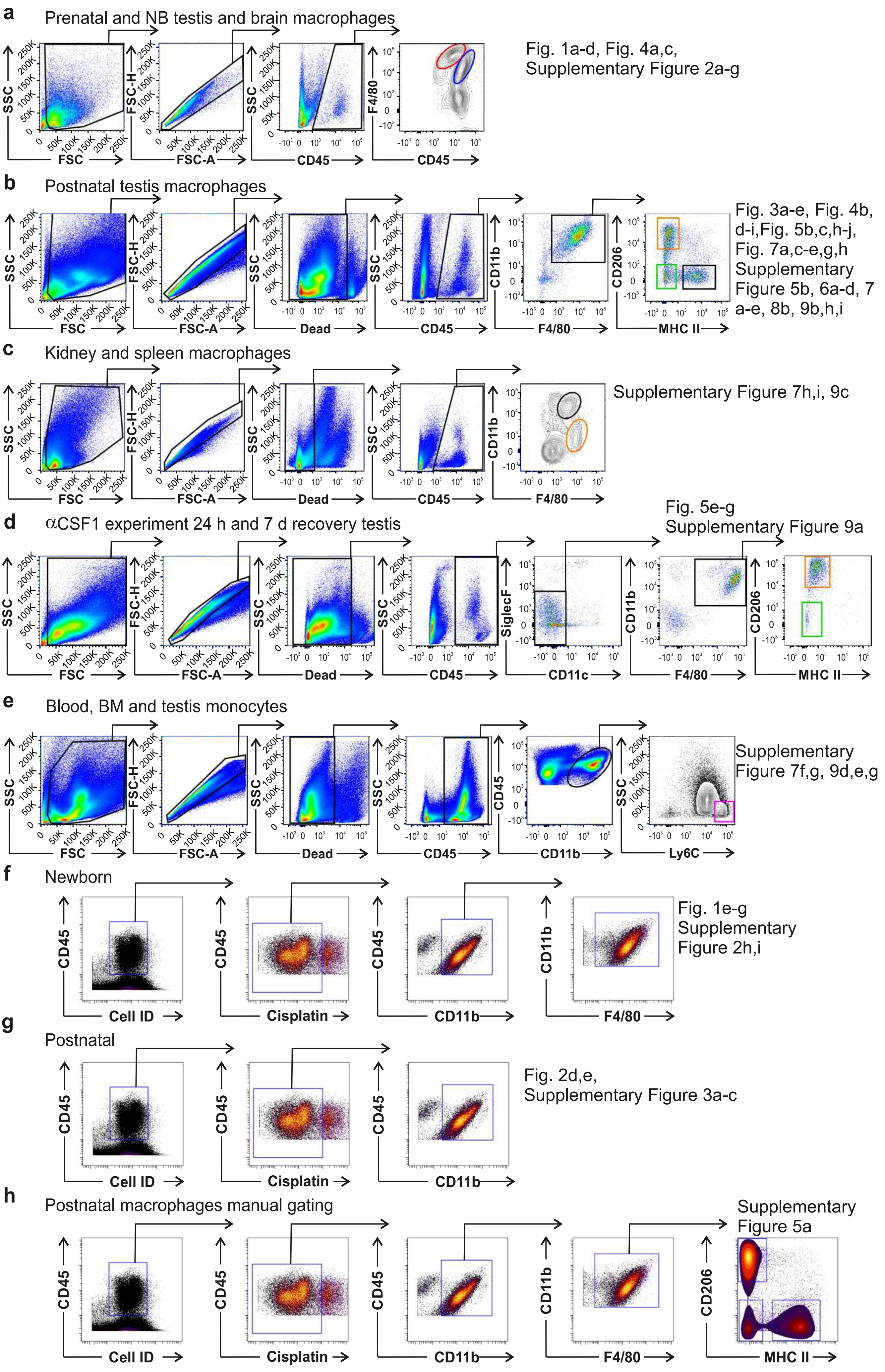

Supplementary Figure 1

Supplementary Figure 1. Gating strategies for flow cytometry

a–**e** FACS gating strategy with representative plots for different leukocyte populations. **a** F4/80^{Hi} and F4/80^{Int} testicular and brain macrophage populations in fetal and newborn (NB) mice. CD45^{+}F4/80^{Hi} macrophages (phenotypically yolk sac-derived) are gated in red, and CD45^{+}F4/80^{Int} macrophages (phenotypically fetal liver-derived) in blue. **b** CD206^{+}MHC II^{-} (orange gate), CD206^{-}MHC II^{-} (green gate), and CD206^{-}MHC II^{+} (black gate) testicular macrophage populations in postnatal mice. **c** Postnatal kidney and spleen F4/80^{Hi} (orange gate) F4/80^{Int} (black gate) macrophages. **d** Testicular macrophages from αCSF1 antibody-treated mice (24 h and 7-day recovery time points only; all other recovery time points from the depletion experiments are gated according to b). **e** Ly6C^{+} monocytes in the blood, bone marrow (BM), and the testis in postnatal mice. All cells are gated for live CD45^{+} events before downstream analysis. **f**–**h** Gating strategy with representative plots for the mass cytometric analyses. Live (Cisplatin^{-}) singlet (Intercalation^{Int}) CD45+ cells positive for (**f**) CD11b and F4/80 in the newborn testis, (**g**) CD11b in postnatal testis and (h) CD11b, F4/80 and CD206/MHC II in the manual gating (for comparison with fluorimetric data) were included in the analyses. The Figure panels, in which the given gating strategy is used, are indicated next to the representative plots.

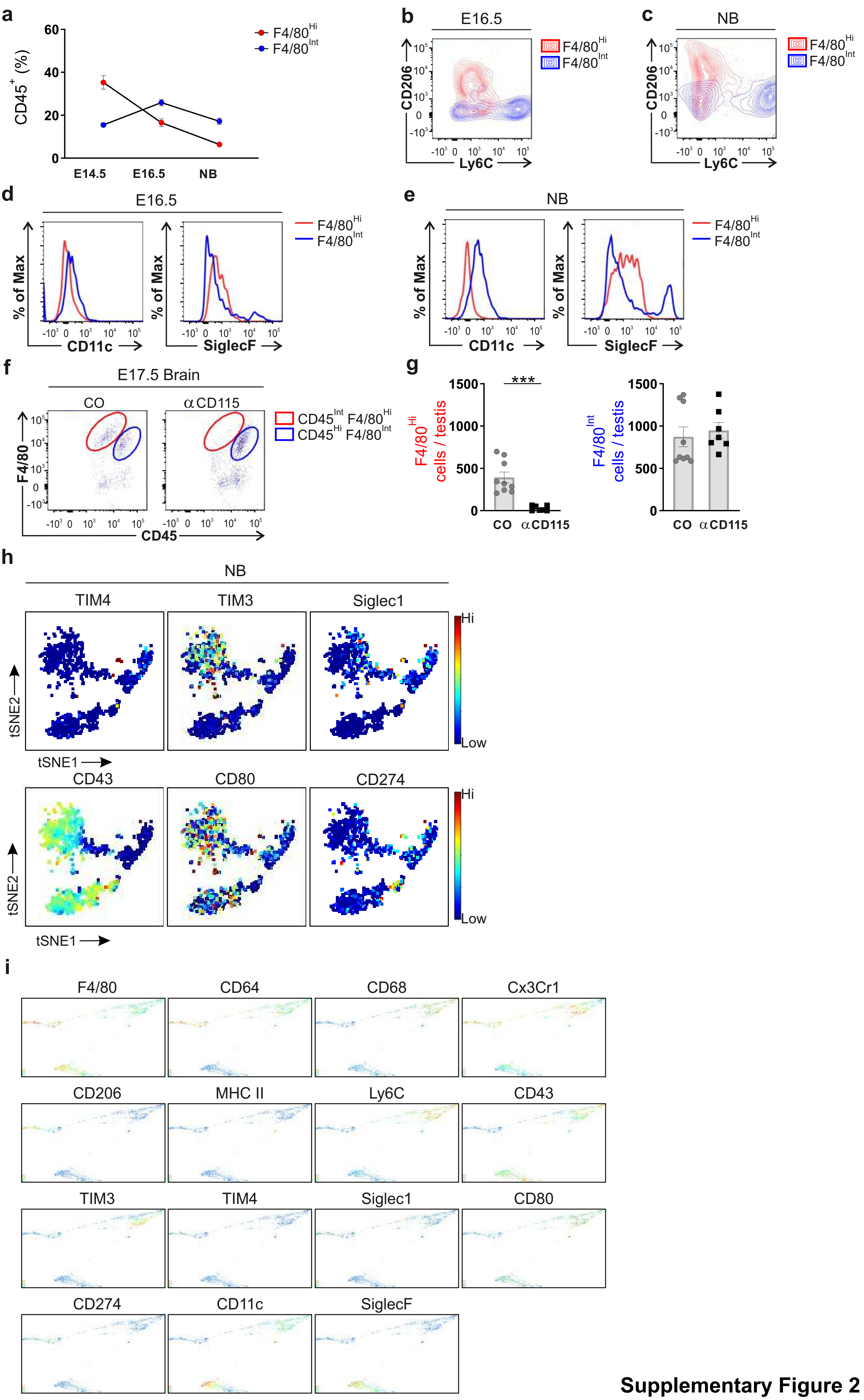

Supplementary Figure 2

Supplementary Figure 2. Analyses of myeloid cells in the testis and other organs of fetal and newborn mice

a Kinetics of testicular F4/80^{Hi} (red) and F4/80^{Int} (blue) macrophage populations in embryonic and newborn (NB) wild type (WT) mice. **b,c** Representative flow cytometry plots of overlaid F4/80^{Hi} (red) and F4/80^{Int} (blue) testicular macrophages in WT E16.5 (**b**) and NB (**c**) mice. **d,e** Representative histograms of CD11c and SiglecF expression in F4/80^{Hi} (red) and F4/80^{Int} (blue) testicular macrophage populations in WT (**d**) E16.5 embryos and (**e**) NB mice. **f** Representative FACS plots of CD45^{Hi} F4/80^{Int} (blue gates) and CD45^{Low} F4/80^{Hi} (red gates) macrophage populations in brains of E17.5 embryos treated with blocking CD115 antibody (AFS98) or isotype-matched control IgG at E6.5. **g** Quantifications of total F4/80^{Hi} and F4/80^{Int} testicular macrophage cell numbers in WT E17.5 embryos treated with blocking CD115 or isotype-matched control IgG at E6.5. In the quantifications (**a, g**), each dot represents a pool of 2–8 testes from 1–4 mice (E14.5, n = 9, and E16.5, n = 8 pools), or both testes of one mouse (E17.5, CO; n = 9, CD115; n = 7 and NB, n = 5 mice). Data are presented as mean ±SEM (***$p<0.001$, Two-tailed Mann–Whitney U test (**g**)). All data are from 2–3 independent experiments. **h** t-SNE maps from mass cytometry analyses displaying the expression of the indicated antigens in randomly sampled live, single CD45$^+$CD11b$^+$F4/80$^+$ cells in the testis of NB WT mice. The scale bar indicates the expression level of a given antigen from low (blue) to high (red). **i** Superimposed expression analyses of the indicated leukocyte markers on unsupervised hierarchical X-shift clustering (nearest neighbor) illustration of live CD45$^+$CD11b$^+$F4/80$^+$ macrophage populations in NB mouse testes (6–10 testes each pooled from 3–5 mice from 2 independent experiments). The expression patterns of the given markers were used for manual designation of the cell clusters in Fig. 1g. Source data are provided as a Source Data file.

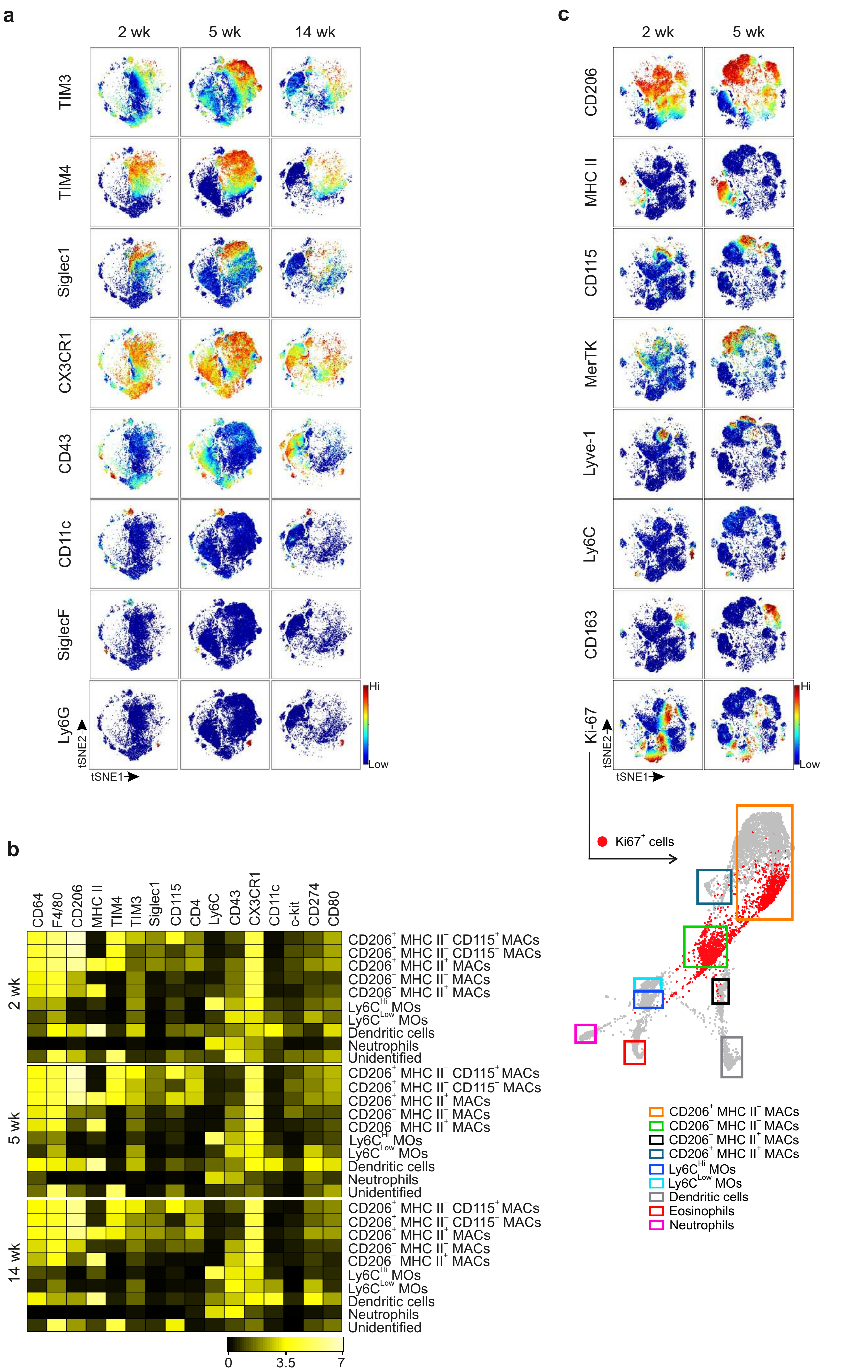

Supplementary Figure 3

Supplementary Figure 3. Single-cell mass cytometry analysis of myeloid cells in the postnatal testis

a t-SNE maps displaying the expression of randomly sampled testicular live single $CD45^{+}CD11b^{+}$ myeloid cells from the testes of 2-, 5- and 14-week-old wild type (WT) mice for the indicated markers. The scale bar indicates the expression level of a given antigen from low (blue) to high (red). **b** Heat map analyses showing the mean expression of indicated markers in FlowSOM meta clusters. The scale bar indicates the expression level of a given antigen from low (black) to high (white) .**c** t-SNE maps displaying the expression of the indicated markers in randomly sampled testicular live, single $CD45^{+}CD11b^{+}$ cells in 2- and 5-week-old WT mice. The scale bar indicates the expression level of a given antigen from low (blue) to high (red). At the bottom is also shown superimposed expression analyses of Ki67 (positive cells shown as red dots) on unsupervised hierarchical X-shift clustering. The colored boxes show manually gated leukocyte populations. Mass cytometry data are from one (**c**) or two (**a**,**b**) independent experiments (*n* = 4 (**a**–**c**) mice per time point).

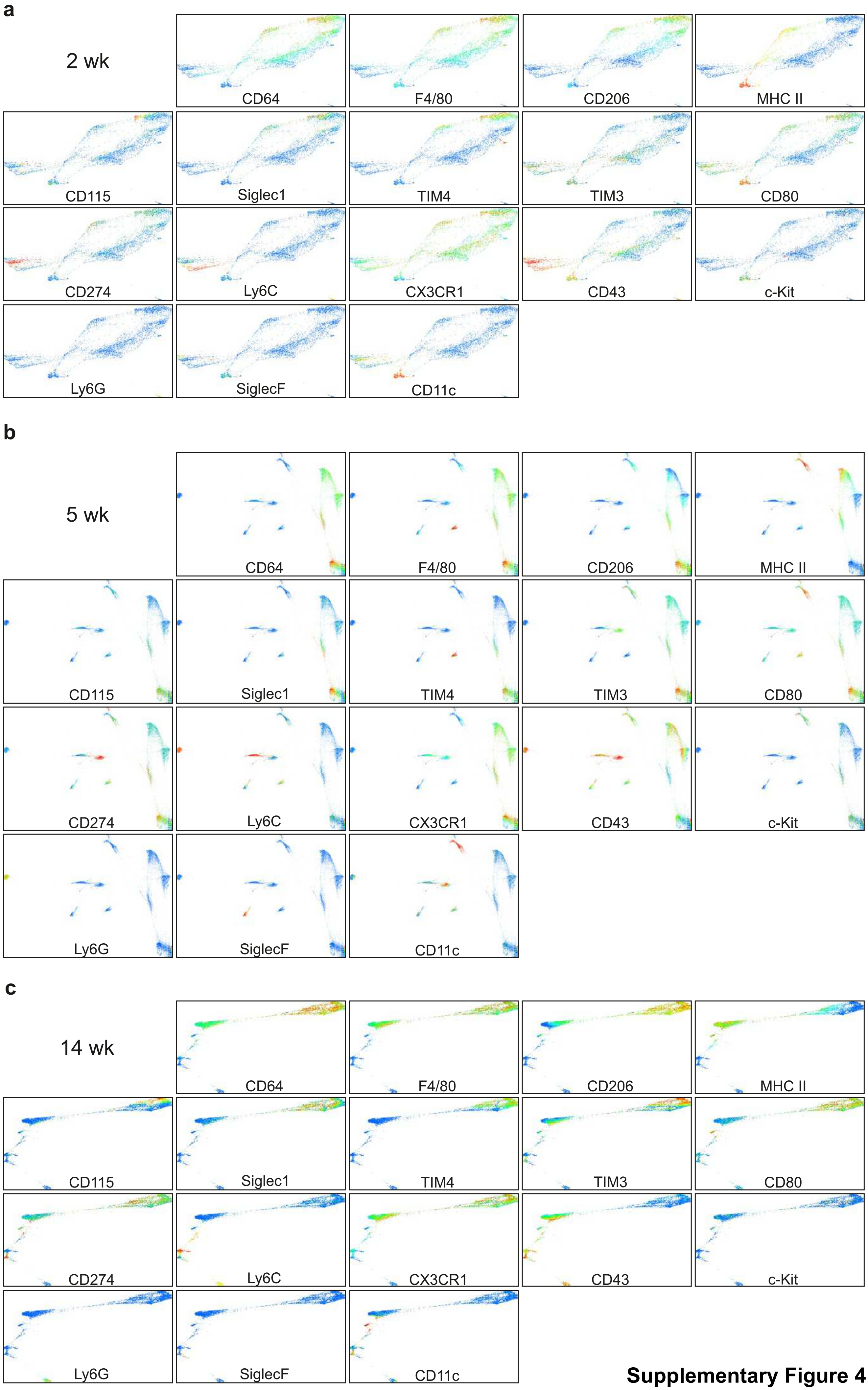

Supplementary Figure 4

Supplementary Figure 4. Trajectory analyses of postnatal testicular macrophages.

a–c Unsupervised hierarchical X-shift clustering (nearest neighbor) of $CD45^{+}CD11b^{+}$ myeloid cells in the testis at indicated time points in wild type mice with superimposed expression analyses of the indicated leukocyte differentiation markers. Color code (blue low to red high) represents expression levels of the indicated markers. The expression patterns of the given markers were used for manual designation of the cell clusters in Fig. 2f. All mass cytometry data are from two independent experiments.

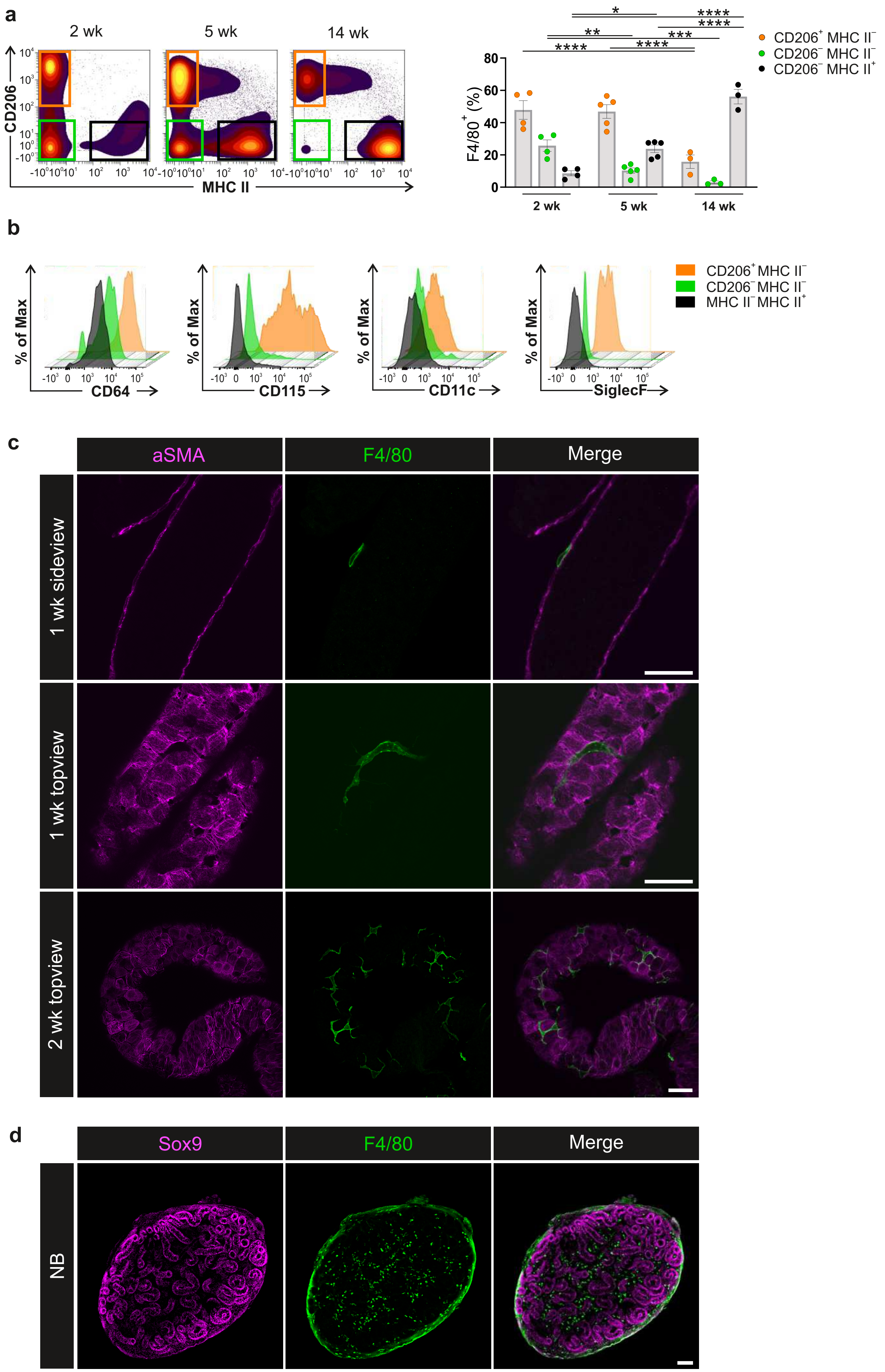

Supplementary Figure 5

Supplementary Figure 5. Analyses of peritubular and interstitial macrophages in the testis

a Manual bi-axial gating and quantification of the mass cytometric data for CD206^{+}MHC II^{-} (orange gates), CD206^{-}MHC II^{-} (green gates), and CD206^{-}MHC II^{+} (black gates) testicular macrophage populations in 2-, 5- and 14-week-old wild type (WT) mice. Each dot represents one mouse (n = 3 (14 wk), n = 4 (2 wk), n = 5 (5 wk) mice). Data are presented as mean ±SEM (* p<0.05, ** p<0.01, ***p<0.001, ****p<0.0001; two-way ANOVA with Bonferroni post hoc test). All data is from 2 independent experiments. **b** Representative histograms from flow cytometric analyses showing CD64, CD115 (CSF1R), CD11c, and SiglecF expression in CD206^{+}MHC II^{-} (orange), CD206^{-}MHC II^{-} (green) and CD206^{-}MHC II^{+} (black) testicular macrophage populations in 5-week-old WT mice. **c** Seminiferous tubule whole-mount stainings of 1- and 2-week-old WT mice with αSMA and F4/80 antibodies. **d** Whole-mount staining of the newborn testis with SOX9 (Sertoli cells) and F4/80 antibodies. Capsular staining is background. **b–d** Shown are representative images from 2–3 mice/time point. Scale bars (**c**,**d**), 50 µm. Source data are provided as a Source Data file.

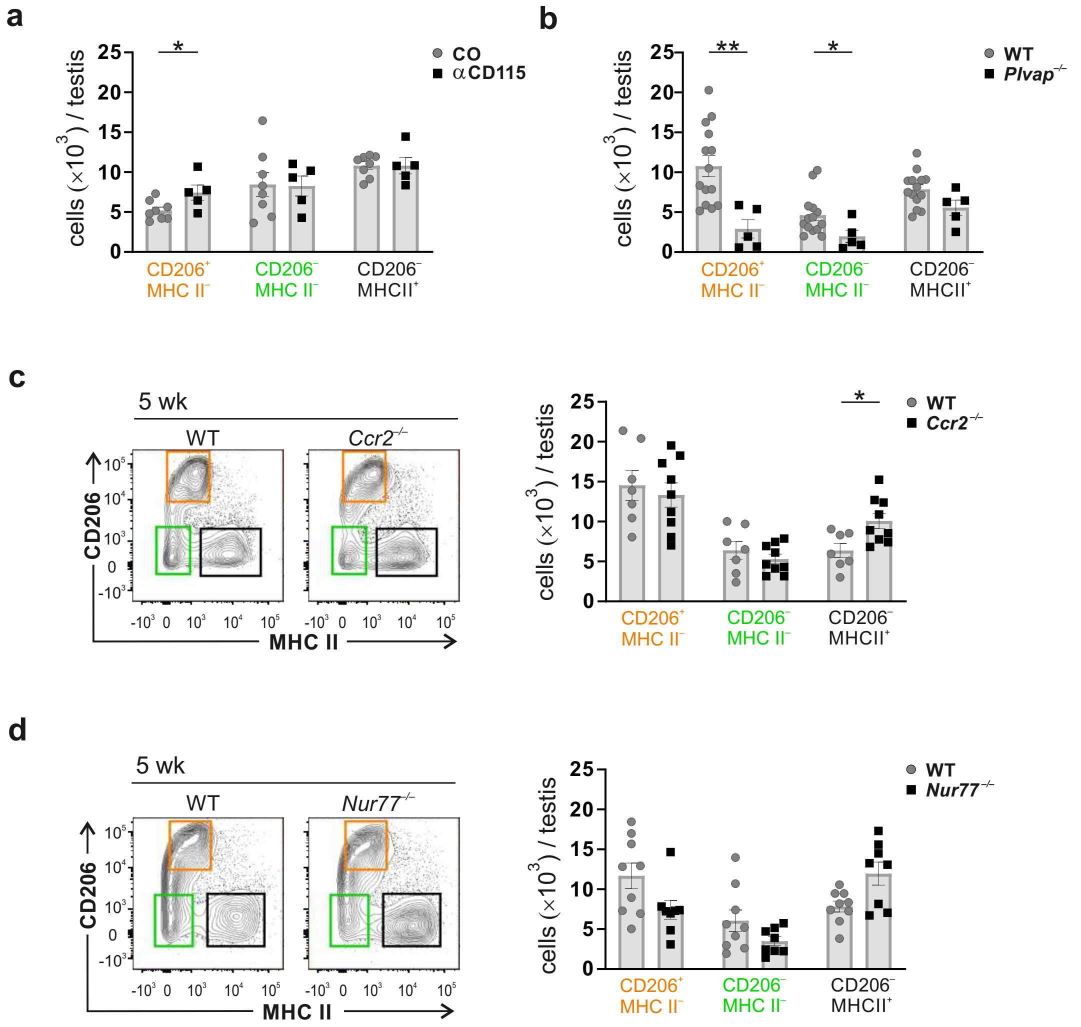

Supplementary Figure 6. The absolute numbers of macrophages in the testis of mice lacking yolk sac-, fetal liver- and adult bone marrow-derived macrophages
a–**d** Total cell numbers and representative dot plots of CD206$^+$MHC II$^-$ (orange), CD206$^-$MHC II$^-$ (green) and CD206$^-$MHC II$^+$ (black) testicular macrophages (**a**) in 5-week-old wild type (WT) mice treated with blocking CD115 antibody or control IgG at E6.5, (**b**) in 5-week-old *Plvap*$^{-/-}$, (**c**) *Ccr2*$^{-/-}$ and (**d**) *Nur77*$^{-/-}$ mice. In the quantifications (**a**–**d**), each dot represents one mouse (n = 5 (**a**: CD115, **b**: *Plvap*$^{-/-}$), n = 7 (**c**:WT), n = 8 (**a**:CO, **d**: *Nur77*$^{-/-}$), n = 9 (**c**: *Ccr2*$^{-/-}$, **d**:WT) or n = 14 (**b**:WT) mice). Data are presented as mean ±SEM (* p<0.05, Two-tailed Mann–Whitney U test (**a**–**c**)). All data are from 2–5 independent experiments. Source data are provided as a Source Data file.

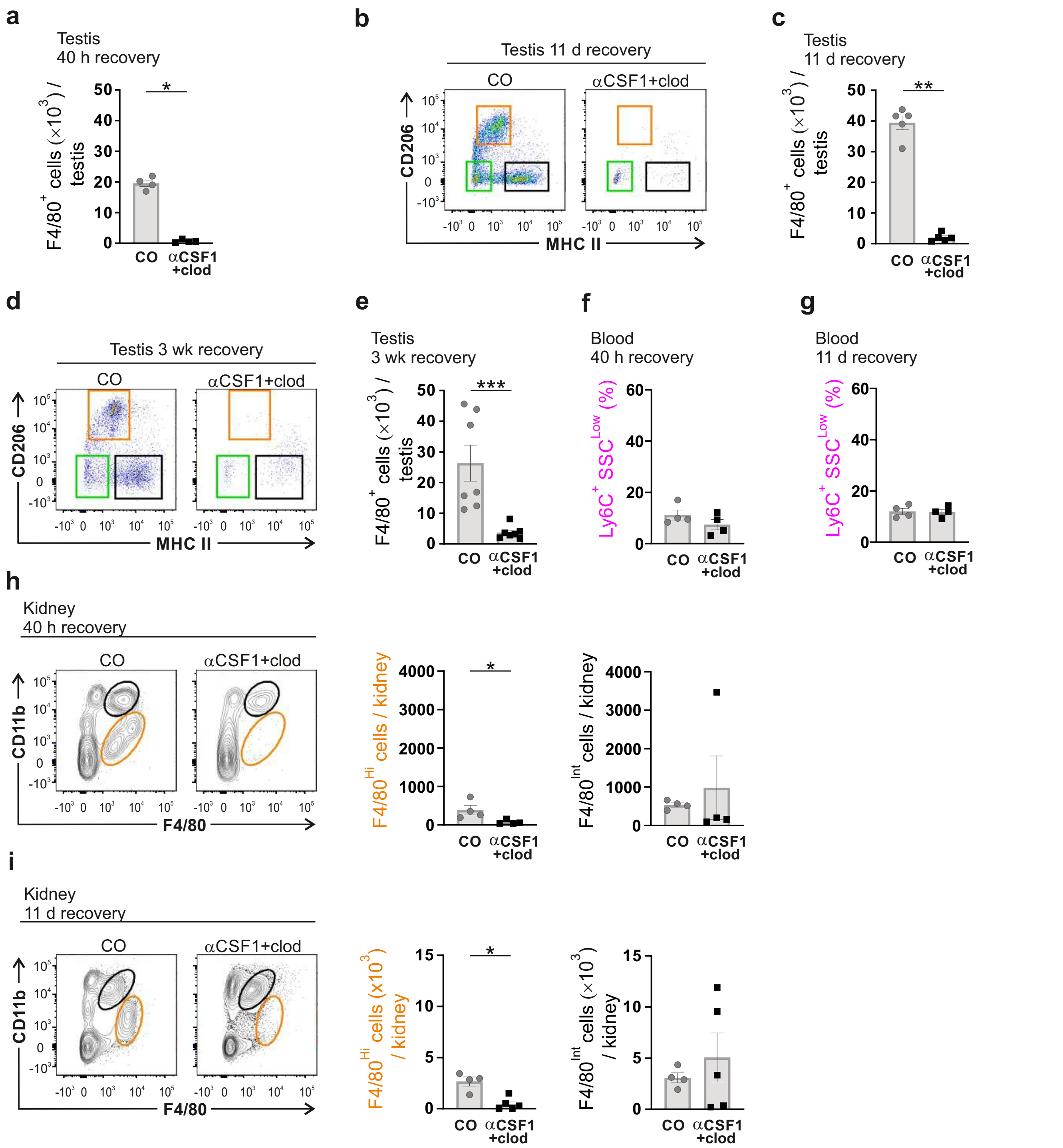

Supplementary Figure 7. Repopulation of macrophage niches emptied with CSF1 antibody and clodronate treatment

a–**i** 10-day-old mice were injected with three alternate cycles of CSF1 (αCSF1) antibody and clodronate (Clod) liposomes (or controls) as detailed in Fig. 5a. **a**–**e** The total numbers of F4/80 testicular macrophages and representative FACS plots after a (**a**) 40-hour, (**b**,**c**) 11-day, and (**d**,**e**) 3-week recovery. **f**,**g** The frequency of blood Ly6C^{+} monocytes after a (**f**) 40-hour and (**g**) 11-day recovery time. **h**,**i** Representative flow cytometry (FACS) plots and total cell numbers of CD11b^{Hi} F4/80^{Int} (black gates) and CD11b^{Int} F4/80^{Hi} (orange gates) renal macrophages after a (**h**) 40-hour and (**i**) 11-day recovery. In the quantifications, each dot represents one mouse (n = 4 (**a**,**f**,**g**,**h**,**i**:CO, **a**,**f**,**g**,**h**:αCSF1+clod), n = 5 (**c**:CO, αCSF1+clod, **i**: αCSF1+clod) or n = 7 (**e**:CO, αCSF1+clod) mice). Data are presented as mean ±SEM (* $p<0.05$, ** $p<0.01$, *** $p<0.001$, Two-tailed Mann–Whitney U test (**c**,**e**,**h**,**i**)). All data are from 2–3 independent experiments. Source data are provided as a Source Data file.

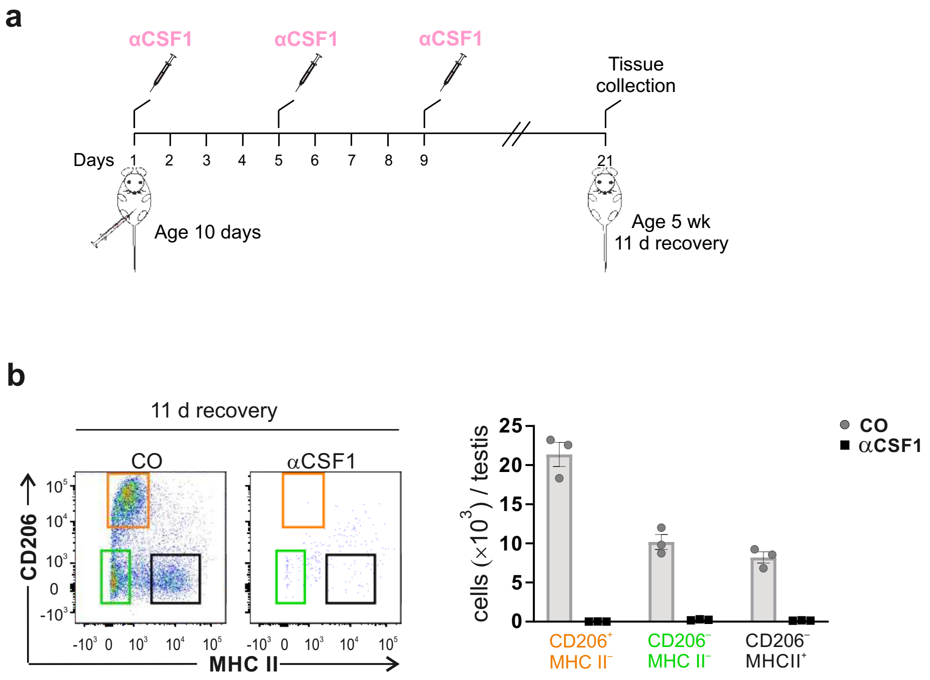

Supplementary Figure 8. Repopulation of macrophage niches emptied with serial CSF1 antibody treatments after the age of 10 days

a Experimental design for depleting macrophages with three subsequent CSF1 (αCSF1) or control antibody (CO) injections starting in 10 d old mice. **b** Representative plots and total numbers of CD206$^+$MHC II$^-$ (orange gates), CD206$^-$MHC II$^-$ (green gates), and CD206$^-$MHC II$^+$ (black gates) testicular macrophages of αCSF1 or CO treated mice in 5-week-old mice after an 11-day recovery. In the quantifications, each dot represents one mouse (*n* = 3 mice per treatment). Data are presented as mean ±SEM. All data are from one experiment. Source data are provided as a Source Data file.

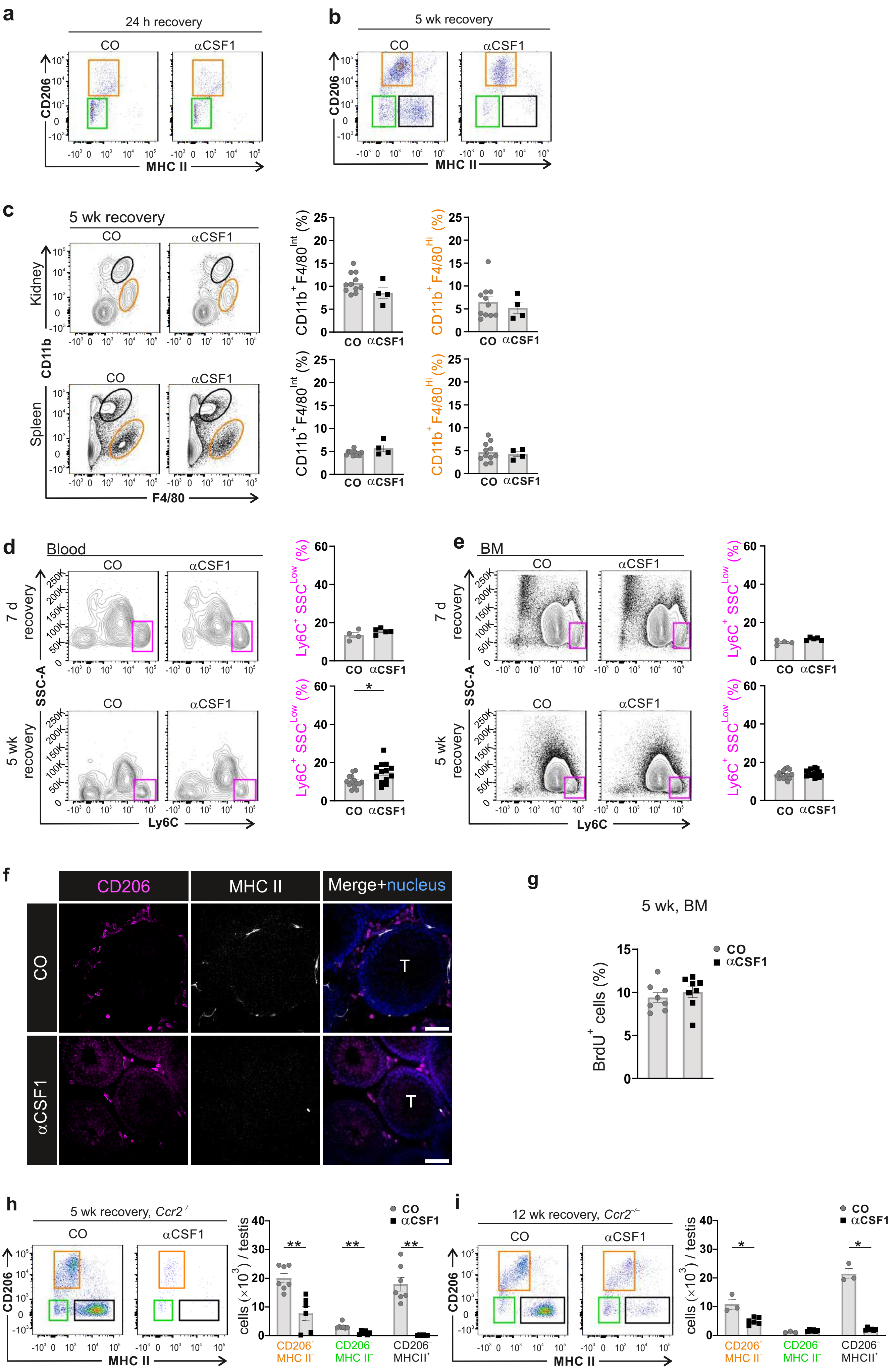

Supplementary Figure 9

Supplementary Figure 9. Validation of a new model using a single CSF1 antibody treatment in newborns for macrophage repopulation studies

a–g Newborn (NB) mice were treated with a single injection of CSF1 (αCSF1) or control (CO) antibody, and analyzed after the indicated recovery periods (as detailed in Fig. 5d) **a,b** Representative FACS plots of testicular macrophages after (**a**) 24-hour, (**b**) and 5-week recovery. **c** Representative FACS plots and the total numbers of kidney and spleen macrophages after a 5-week recovery. **d,e** Representative FACS plots and the total numbers of Ly6C-positive monocytes in (**d**) blood and (**e**) bone marrow after a 7-day and 5-week recovery. **f** Maximum intensity projection of a vibratome section of the testis stained for CD206 and MHC II after a 5-week recovery time. T, tubules. Scale bars, 50 µm. **g** *In vivo* BrdU-labeling of the bone marrow leukocytes at a 5-week recovery time point. **h,i** Newborn *Ccr2*$^{-/-}$ mice were treated with a single injection of CSF1 (αCSF1) or control (CO) antibody. Representative FACS plots and the total numbers of testicular macrophages after a (**i**) 5-week and (**j**) 12-week recovery. In the quantifications, each dot represents one mouse (n = 3 (**i**:CO), n = 4 (**c**: αCSF1, **d**,**e**:7d;CO), n = 5 (**d**,**e**:7d; αCSF1), n = 6 (**h**,**i**:αCSF1), n = 7 (**h**:CO), n = 8 (**g**:CO, αCSF1), n = 11(**c**:CO), n = 13 (**d**:5wk; αCSF1) or n = 14 (**d**:5wk;CO, e:CO, αCSF1) mice). Data are presented as mean ±SEM (* $p<0.05$, ** $p<0.01$, Two-tailed Mann–Whitney U test (**h,i**)). All data are from 2-5 independent experiments. Source data are provided as a Source Data file.

a

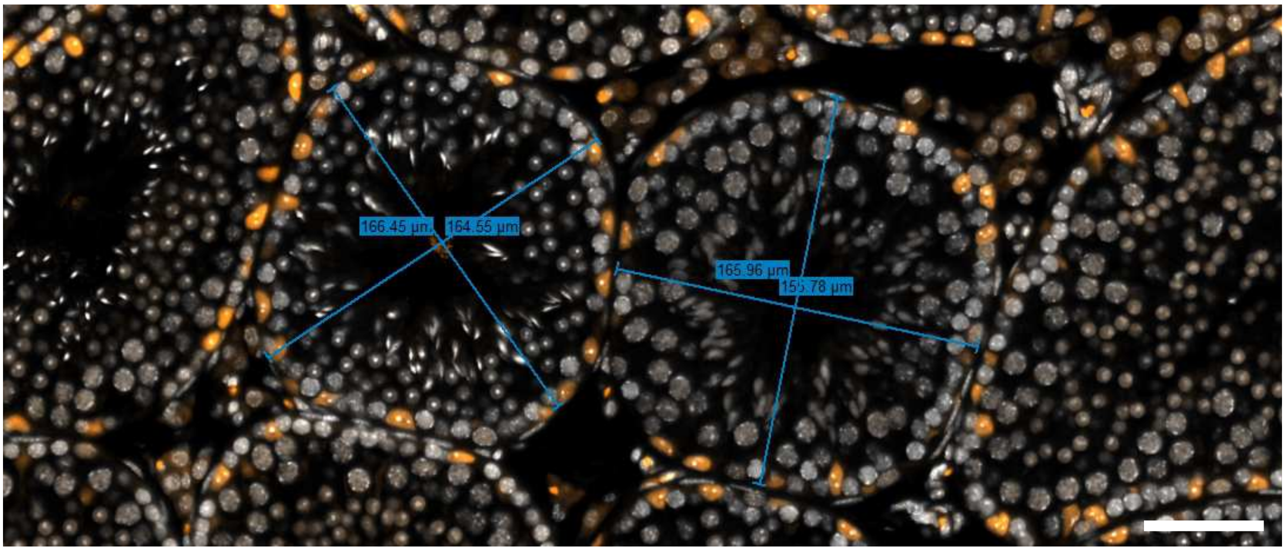

b

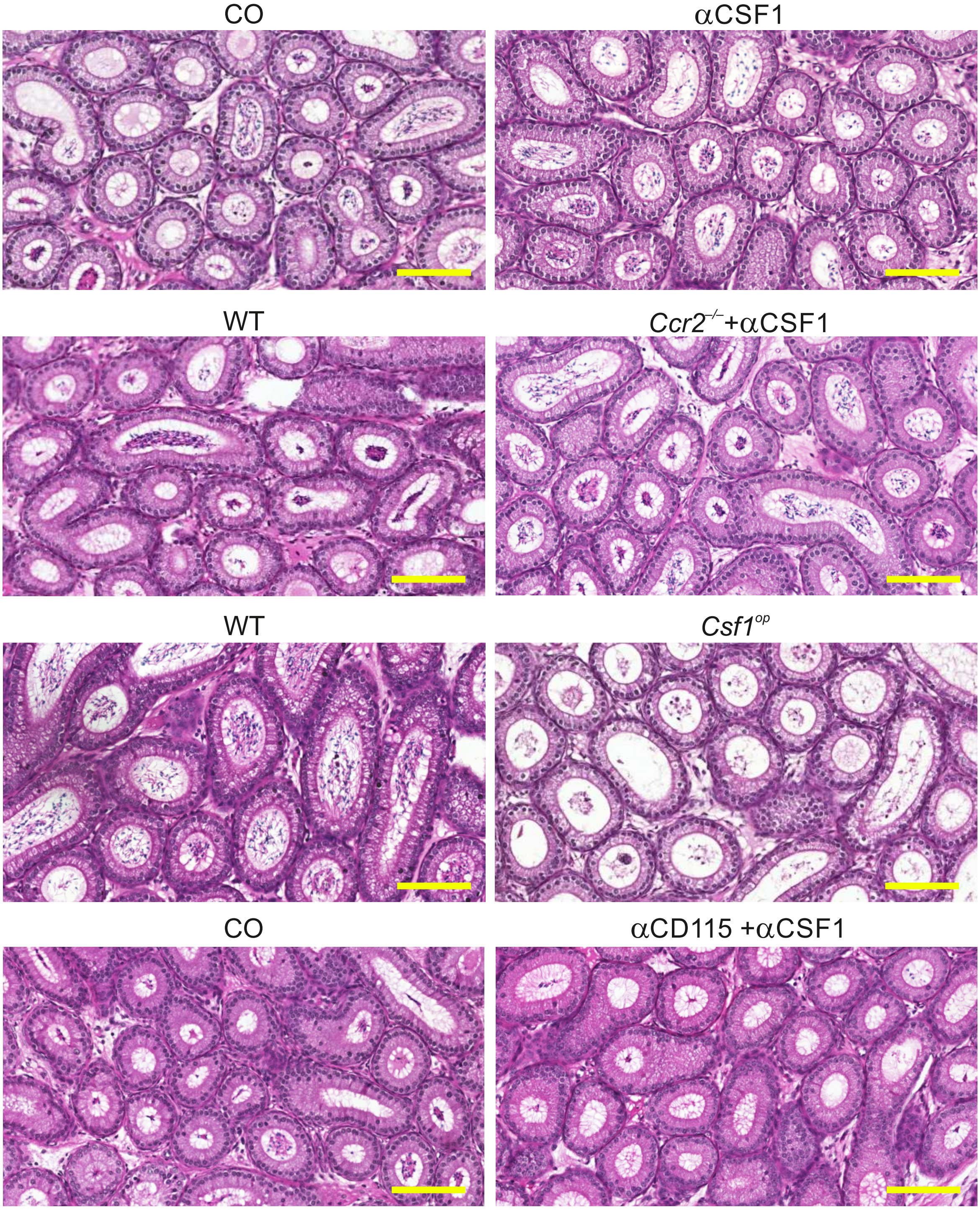

Supplementary figure 10

Supplementary Figure 10. Histology of testis and epididymis

a The diameter of a seminiferous tubule is recorded as an average of two perpendicular diameter measurements from a rounded tubular cross-section in SOX9 antibody and DAPI stained sections. Scale bar, 50 µm. **b** HE-stainings of the caput epididymis in 5-week-old mice, in which macrophages have been depleted using the indicated protocols: wild-type (WT) mice treated with CSF1 antibody at birth (αCSF1), *Ccr2*$^{-/-}$ mice treated with CSF1 antibody at birth (*Ccr2*$^{-/-}$+αCSF1), *Csf1*^{op} mice, and WT mice treated with CD115 antibody at E6.5 and with CSF1 antibody at birth and the age of 2 week (αCD115+αCSF1), and their appropriate controls (WT, wild-type mice; CO, isotype-matched control antibody-treated mice). Note the presence or absence of sperm cells in the lumen. Representative images from 3 mice per experimental group are shown. Scale bars, 100 µm.

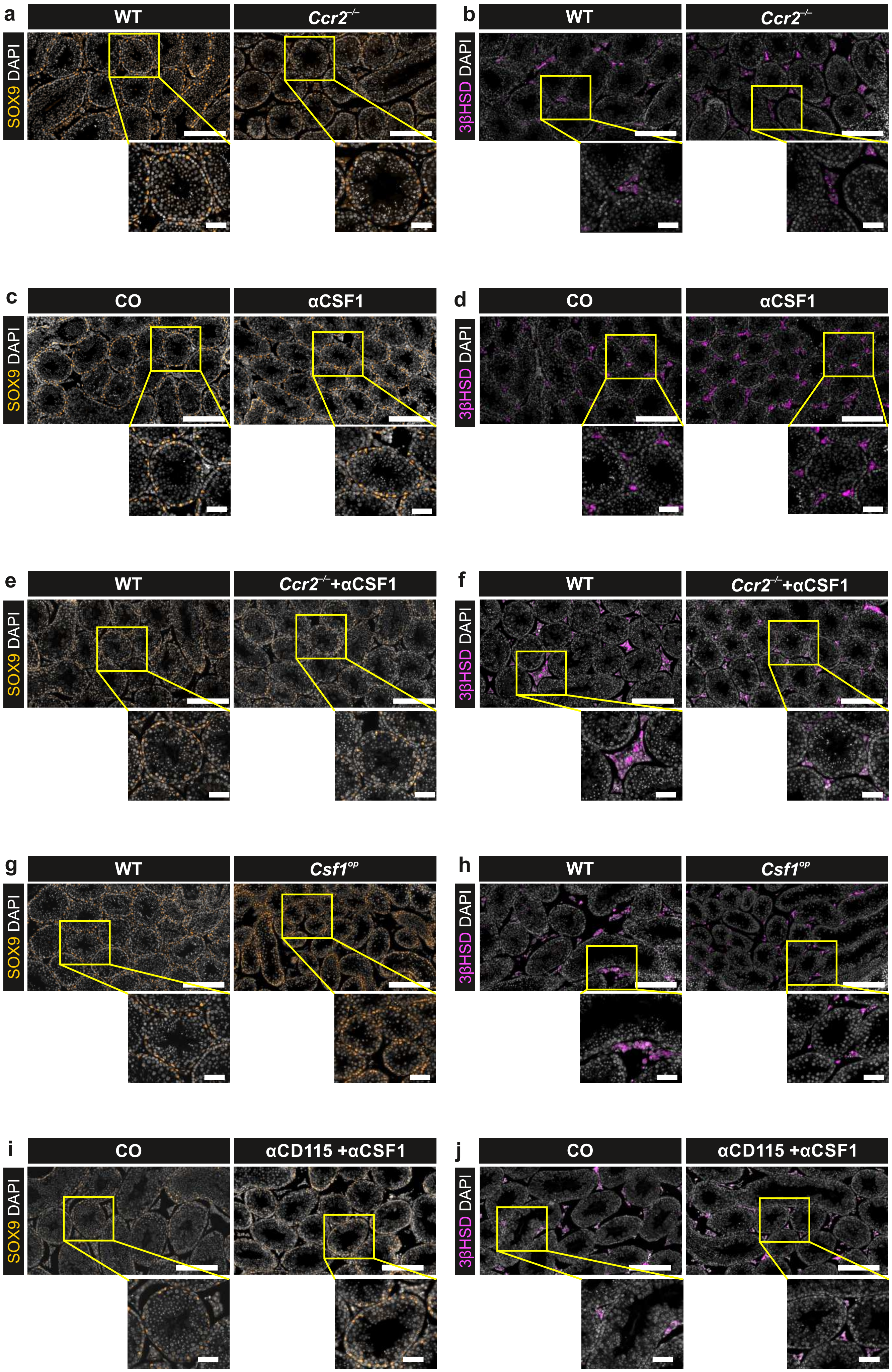

Supplementary Figure 11

Supplementary Figure 11. Histomorphometry of the testicular somatic cell populations

a–**j** Immunohistochemical analysis of Sertoli cells expressing SOX9 (left column) and Leydig cells expressing 3βHSD (right column) in the testis of 5-week-old (**a,b**) *Ccr2*$^{-/-}$ mice, (**c,d**) CSF1 (αCSF1) (or control antibody (CO); single injection after birth) treated wild type (WT) mice, (**e,f**) CSF1 (αCSF1) (or control (CO) antibody; single injection after birth) treated *Ccr2*$^{-/-}$, (**g,h**) *Csf1*^{op} mice and (**i,j**) CD115 (E6.5)+ CSF1 (newborn and 2 wk) or control (CO) antibody-treated WT mice. Scale bars (**a**–**j**), 200 µm, and 50 µm in inserts. Representative images from at least 3 mice per experimental group are shown.

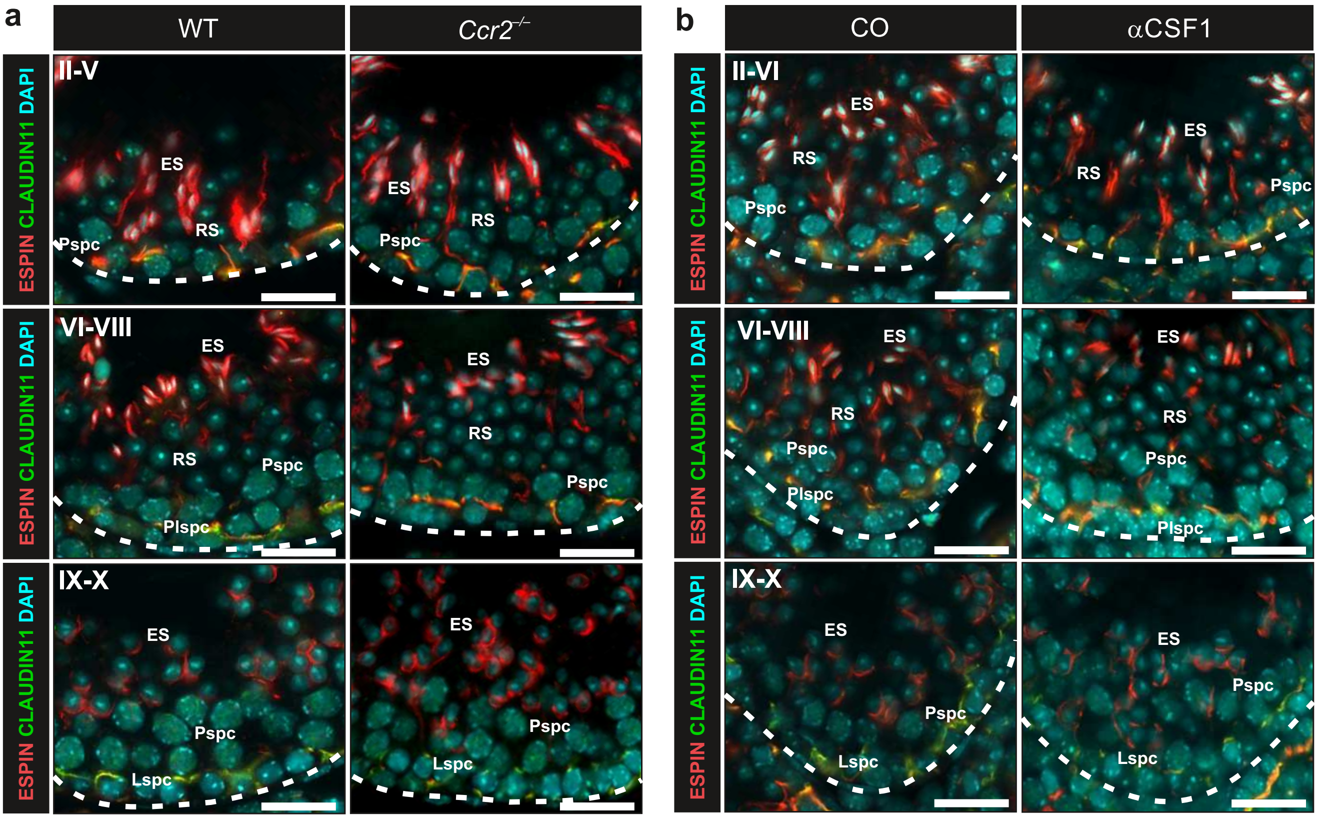

Supplementary Fig. 12. Blood-testis barrier and spermatocyte development in macrophage-depleted mice.
a–**b** Immunohistological analyses of blood-testis barrier in the testis of 5-week-old (**a**) *Ccr2*$^{-/-}$ mice, (**b**) wild type (WT) mice treated with CSF1 antibody at birth (αCSF1). Representative images (from at least 3 mice per experimental group) of the testis sections (II-V, VI-VIII and IX) representing specific stages of the seminiferous epithelial cycle immunostained with CLAUDIN 11 (to detect tight junctions between Sertoli cells) and ESPIN (to detect actin bundles on the Sertoli cell-side of the basal and apical ectoplasmic specializations) antibodies. Nuclei were stained with DAPI. Dashed line: tubule boundary, ES: elongating spermatids, RS: round spermatids, Pspc: pachytene spermatocytes, Lspc: leptotene spermatocytes, Plspc: preleptotene spermatocytes. Scale bars (**a**,**b**), 25 μm.

Supplementary Table 1. Antibodies used in this study

**Antibodies**			**Dilutions/Concentrations**			
			**Flow**	**CyTOF**	**Immunohisto.**	***In Vivo***
Anti APC-176Yb	Fluidigm	cat# 3176007C		1:200		
Anti FITC-144Nd	Fluidigm	cat# 3144006C		1:200		
Anti human CLDN11	Santa Cruz	cat# sc-25711			1:200	
Anti human SOX9	EMD Millipore	cat# AB5535			1:400	
Anti mouse aSMA-A488	Abcam	cat# ab184675			1:500	
Anti mouse B220-160Gd	Fluidigm	cay# 3160012C		1:200		
Anti mouse B220-159Tb	DVS Sciences	cat# 3159015C		1:200		
Anti-BrdU-FITC	BD Biosciences	cat# 559619		1:200		
Anti mouse CD4-172Yb	Fluidigm	cat# 3172003C		1:200		
Anti mouse CD8a-168Er	Fluidigm	cat# 3168003C		1:200		
Anti mouse CD11b-148Nd	Fluidigm	cat# 3148003C		1:200		
Anti mouse CD11b-PE	BD Biosciences	cat# 553311	1:400			
Anti mouse CD11b-APC-Cy7	BD Biosciences	cat# 557647	1:400			
Anti mouse CD11b-BB515	BD Biosciences	cat# 564454	1:400			
Anti mouse CD11b-BV786	BD Biosciences	cat# 740861	1:400			
Anti mouse CD11c-142Nd	Fluidigm	cat# 3142003C		1:200		
Anti mouse CD11c-BV711	BioLegend	cat# 117349	1:200			
Anti mouse CD16/CD32 (2.4G2)	Bio X Cell	cat# CUSTOM24G2	1.5:100			
Anti mouse CD31 (MEC13.3)	BD Biosciences	cat# 553370			10 µg/ml	
Anti mouse CD45-147Sm	Fluidigm	cat# 3147003C		1:200		
Anti mouse CD45-PerCP-Cy5.5	BD Biosciences	cat# 550994	1:200			
Anti mouse CD64-151Eu	Fluidigm	cat# 3151012C		1:200		
Anti mouse CD64-PE	BioLegend	cat# 139304	1:200			
Anti-mouse CD68-FITC	BioLegend	cat# 137005		1:200		
Anti mouse CD80-171Yb	Fluidigm	cat# 3171008C	1:200			
Anti mouse CD115-PE-Cy7	eBioscience	cat# 25-1152-805	1:400			

Anti mouse CD117-173Yb	Fluidigm	cat# 3173004C		1:200		
Anti mouse CD163	BioLegend	cat# 155302		1:100		
Anti mouse CD206-169Tm	Fluidigm	cat# 3169021C		1:200		
Anti mouse CD206-BV650	BioLegend	cat# 141723	1:200			
Anti mouse CD206-A488	BioRad	cat# MCA2235A488T			10 µg/ml	
Anti mouse CD206-A647	BD Biosciences	cat# 565250			8 µg/ml	
Anti mouse CD274-153Eu	Fluidigm	cat# 3153016C		1:200		
Anti mouse CSF1 (5A1)	Bio X Cell	cat# BE0204				0.5, 0.25 or 0.15 mg/injection
Anti mouse CSF1R (AFS98)	Bio X Cell	cat# BE0213				3 mg/ injection
Anti mouse CX3CR1-164Dy	Fluidigm	cat# 3164023C		1:200		
Anti mouse F4/80-159Tb	Fluidigm	cat# 3159009C		1:200		
Anti mouse F4/80-146Nd	Fluidigm	cat# 3146008C		1:200		
Anti mouse F4/80-A488	eBioscience	cat# 53-4801-82	1:200			
Anti mouse F4/80 (CI:A3-1)	Bio-Rad	cat# MCA497R			10 µg/ml	
Anti mouse F4/80 (CI:A3-1)	Bio X Cell	cat# BE0206			10 µg/ml	
Anti mouse F4/80-A647	Bio-Rad	cat# MCA497A647	1:100			
Anti mouse Hsd3b	TransGenic Inc	cat# KO607			1:200	
Anti mouse IgG1 (HRPN)	Bio X Cell	cat# BE0088				0.5, 0.25 or 0.15 mg/injection
Anti mouse IgG2a (2A3)	Bio X Cell	cat# BE0089				3 mg/injection
Anti mouse Ki-67	eBioscience	cat# 11-5698-82			1:200	
Anti mouse Ki-67-161Dy	Fluidigm	cat# 3161007C		1:200		
Anti mouse Ly6C-150Nd	Fluidigm	cat# 3150010C		1:200		
Anti mouse Ly6C-162Dy	Fluidigm	cat# 3162014C		1:200		
Anti mouse Ly6C-BV421	BD Biosciences	cat# 562727	1:200			
Anti mouse Ly6G-141Pr	Fluidigm	cat# 3141008C		1:200		
Anti mouse Ly6G-BV510	BioLegend	cat# 127633	1:200			
Anti mouse LYVE-1-PE	R&D Systems	cat# FAB2125P		1:200		

Anti mouse MerTK-FITC	BioLegend	cat# 151503		1:200		
Anti mouse MerTK-PE	BioLegend	cat# 151505	1:200			
Anti mouse MHC II-174Yb	Fluidigm	cat# 3174003C		1:200		
Anti mouse MHC II-PE	BD Biosciences	cat# 557000	1:400			
Anti mouse MHC II-BV711	BD Biosciences	cat# 563414	1:400			
Anti mouse MHC II-A647	BioLegend	cat# 107618			10 μg/ml	
Anti mouse MHC II-A488	eBioscience	cat# 25-5321-82	1:400			
Anti mouse Siglec-1-170Er	Fluidigm	cat# 3170018C		1:200		
Anti mouse Siglec-F-APC	BioLegend	cat# 155507	1:200	1:200		
Anti mouse Siglec-F-PE-CF594	BD Biosciences	cat# 562757	1:200	1:100		
Anti mouse TER-119-154Sm	Fluidigm	cat# 3154005C		1:200		
Anti mouse Tim-3-162Dy	Fluidigm	cat# 3162029C		1:200		
Anti mouse Tim-4	BioLegend	cat# 130002		1:100		
Anti PE-165HO	Fluidigm	cat# 3165015C		1:200		
Anti-FITC-144Nd	Fluidigm	cat# 3176007C		1:200		
Anti-APC_178Yb	Fluidigm	cat# 3144006C		1:200		
Anti rabbit IgG-A546	ThermoFisher Scientific	cat# A11035			1:500	
Anti rabbit IgG-A594	ThermoFisher Scientific	cat# A21207			1:500	
Anti rat Espin	BD Biosciences	cat# 611656			1.500	
Anti rat IgG-A488	ThermoFisher Scientific	cat# A11006			1:500	
Anti rat IgG-A488	ThermoFisher Scientific	cat# A21208			1:400	
Anti rat IgG-A546	ThermoFisher Scientific	cat# A11081			1:500	
Anti rat IgG-A594	ThermoFisher Scientific	cat# A11007			1:400	
