# Supplementary material for: Generation, localization and functions of macrophages during the development of testis
Source: Nat Commun. 2020 Sep 1;11:4375. doi: 10.1038/s41467-020-18206-0 (PMC7463013; doi:10.1038/s41467-020-18206-0)
Supplement: Supplementary file 3 — Reporting Summary [file 41467_2020_18206_MOESM3_ESM.pdf]

nature research

Corresponding author(s): Marko Salmi, Pia Rantakari

Last updated by author(s): Aug 3, 2020

Reporting Summary

Nature Research wishes to improve the reproducibility of the work that we publish. This form provides structure for consistency and transparency in reporting. For further information on Nature Research policies, see Authors & Referees and the Editorial Policy Checklist .

Statistics

For all statistical analyses, confirm that the following items are present in the figure legend, table legend, main text, or Methods section.

n/a	Confirmed	
☐	☒	The exact sample size (*n*) for each experimental group/condition, given as a discrete number and unit of measurement
☐	☒	A statement on whether measurements were taken from distinct samples or whether the same sample was measured repeatedly
☐	☒	The statistical test(s) used AND whether they are one- or two-sided<br>*Only common tests should be described solely by name; describe more complex techniques in the Methods section.*
☒	☐	A description of all covariates tested
☒	☐	A description of any assumptions or corrections, such as tests of normality and adjustment for multiple comparisons
☐	☒	A full description of the statistical parameters including central tendency (e.g. means) or other basic estimates (e.g. regression coefficient) AND variation (e.g. standard deviation) or associated estimates of uncertainty (e.g. confidence intervals)
☐	☒	For null hypothesis testing, the test statistic (e.g. *F*, *t*, *r*) with confidence intervals, effect sizes, degrees of freedom and *P* value noted<br>*Give P values as exact values whenever suitable.*
☒	☐	For Bayesian analysis, information on the choice of priors and Markov chain Monte Carlo settings
☒	☐	For hierarchical and complex designs, identification of the appropriate level for tests and full reporting of outcomes
☒	☐	Estimates of effect sizes (e.g. Cohen's *d*, Pearson's *r*), indicating how they were calculated

Our web collection on statistics for biologists contains articles on many of the points above.

Software and code

Policy information about availability of computer code

Data collection

Spinning disk confocal images were acquired with Slide Book 6 software (Intelligent Imaging Innovations), confocal microscope images were acquired with Zen 2010 software (Zeiss), immunofluorescence and bright field images were acquired with The 3DHISTECH software (3DHISTECH Ltd.). Flow cytometry data were acquired with FACSDiVaTM software version 8 (BD Bioscience). Mass cytometry data were acquired with CyTOF 6.7 system control software (Fluidigm).

Data analysis

Confocal microscopy data were analyzed with ImageJ software. Slidescanner data (brightfield and immunofluorescence) were analyzed with Pannoramic viewer version 1.15 or CaseViewer version 2.4 software(3DHISTECH Ltd). Flow cytometry data were analyzed with FlowJo version 10 (TreeStar). Mass cytometry data were analyzed with Cytobank (https://www.cytobank.org) or with VorteX clustering environment (https://github.com/nolanlab/vortex/tag/29-Jun-2017; Published in Nat Methods. 2016 June ; 13(6): 493-496. doi:10.1038/nmeth.3863). VorteX clustering visualization were produced with Gephi 0.9.1 (https://gephi.org). Statistical analyses were done using GraphPad Prism 8 (GraphPad Software, Inc.). Custom algorithms or software were not used in this study.

For manuscripts utilizing custom algorithms or software that are central to the research but not yet described in published literature, software must be made available to editors/reviewers. We strongly encourage code deposition in a community repository (e.g. GitHub). See the Nature Research guidelines for submitting code & software for further information.

Data

Policy information about availability of data

All manuscripts must include a data availability statement. This statement should provide the following information, where applicable:

- Accession codes, unique identifiers, or web links for publicly available datasets
- A list of figures that have associated raw data
- A description of any restrictions on data availability

All materials used in this study are available commercially or from the authors. The sources for the materials are in the Materials and Methods section. Supplementary Information file is available in online version of this article. All raw data that support the findings of this study are available from the corresponding authors upon reasonable request. Mass cytometry data files are also available from FlowRepository (http://flowrepository.org, FR-FCM-Z2RQ). The source data

underlying Figures 1a, d, 2d, e, 3a–e, 4a–i, 5c, f–j, 6a, b, e, f, 7a,c–e, g, h and Supplementary Figures 2a, g, 5a, 6a–d, 7a, c, e–i, 8b, and 9c–f, h–j are provided as a Source Data file with this paper.

Field-specific reporting

Please select the one below that is the best fit for your research. If you are not sure, read the appropriate sections before making your selection.

- [x] Life sciences
- [ ] Behavioural & social sciences
- [ ] Ecological, evolutionary & environmental sciences

For a reference copy of the document with all sections, see nature.com/documents/nr-reporting-summary-flat.pdf

Life sciences study design

All studies must disclose on these points even when the disclosure is negative.

Sample size	No specific statistical test was used to determine the sample size.
Data exclusions	All data from experiments in which the positive and/or negative controls worked appropriately were included in the study. (If the positive and/or negative controls did not work, the whole experiment was excluded from the analyses; Fig. 4c,d).
Replication	All data are at least from 2-13 mice. The exact n-numbers are indicated in each Figure legend.
Randomization	Littermate wild-type mice of the same sex were randomly assigned to the experimental groups. When gene-modified mice were used, age- , sex- and strain-matched wild-types were used as controls.
Blinding	The investigators were not blinded to mouse allocation during experiments and outcome assessments.<br>Data collection could not be done in a blinded fashion as many used genetically modified mice can be visually separated from the littermates or control mice. In analysis of the experiments using the expression of the YFP reporter, blinding was not relevant as the YFP expression could be identified in FACS and imaging based assays.

Reporting for specific materials, systems and methods

We require information from authors about some types of materials, experimental systems and methods used in many studies. Here, indicate whether each material, system or method listed is relevant to your study. If you are not sure if a list item applies to your research, read the appropriate section before selecting a response.

Materials & experimental systems

n/a	Involved in the study	
[ ]	[x]	Antibodies
[x]	[ ]	Eukaryotic cell lines
[x]	[ ]	Palaeontology
[ ]	[x]	Animals and other organisms
[x]	[ ]	Human research participants
[x]	[ ]	Clinical data

Methods

n/a	Involved in the study	
[x]	[ ]	ChIP-seq
[ ]	[x]	Flow cytometry
[x]	[ ]	MRI-based neuroimaging

Antibodies

Antibodies used	All antibodies (clones, host species, isotypes, fluorochrome-conjugates, vendors and concentrations/ dilutions) have been described in Supplementary Table 1.
Validation	The primary antibodies have been validated for immunohistochemistry on frozen sections or for the flow cytometry or mass cytometry by our previous published work (Endocrinology 160:5 (2019), Nat Commun 10, 281 (2019), Eur J Immunol. May 27 (2020)) or by the specificity controls (isotype controls) or by the commercial vendors. Validation information is provided in the respective manufacturer's website.

Animals and other organisms

Policy information about studies involving animals; ARRIVE guidelines recommended for reporting animal research

Laboratory animals	Plvap-/- mice have been previously described (Rantakari et al., 2015; Rantakari et al., 2016b). Ccr2–/– (stock 004999), Nur77–/– (stock 006187), R26R-EYFP (stock 006148), CX3CR1CreERT2 (stock 020940), Csf1rMer-iCre-Mer (stock 019098) and Csf1op (stock 000231) mice were purchased form Jackson Laboratories. C57BL/6J and C57BL/6N mice were purchased from Janvier labs. Wild type male mice were used at age of E14.5, E16.5, E17.5, new born, 2d, 7d, 1wk,2wk, 3wk, 5wk, 8wk, 12wk and 14wk. Csf1rMer-iCre-Mer/R26R-EYFP male mice were used at age of E16.5 and 5wk. CX3CR1CreERT2/R26R-EYFP male mice were used at age of new born, 5wk and 10wk. Plvap-/- , Csf1op, Ccr2–/– and Nur77–/– male mice were used at age of 5wk.
Wild animals	Not used
Field-collected samples	Not used
Ethics oversight	Animal experiments were approved by the National Animal Experiment Board in Finland (Animal license number 6211/04.10.07/2017). They were carried out in adherence with the rules and regulations of the Finnish Act on Animal Experimentation (497/2013). All the animal experiments were performed in accordance to the 3R-principle.

Note that full information on the approval of the study protocol must also be provided in the manuscript.

Flow Cytometry

Plots

Confirm that:

- [x] The axis labels state the marker and fluorochrome used (e.g. CD4-FITC).
- [x] The axis scales are clearly visible. Include numbers along axes only for bottom left plot of group (a 'group' is an analysis of identical markers).
- [x] All plots are contour plots with outliers or pseudocolor plots.
- [x] A numerical value for number of cells or percentage (with statistics) is provided.

Methodology

Sample preparation	Fetal testes were dissociated to flow cytometry buffer (2 % fetal calf serum (FCS; v/v) and 0.05 % NaN3 (v/v) in phosphate-buffered saline (PBS)) by pipetting. Embryonic brain was minced and digested with 50 µg/ml DNase 1 (Roche, cat. 10104159001) and 1 mg/ml Collagenase D (Roche, cat. 1108886601) in Hanks' solution at +37 °C for 45 min. Leukocytes were then separated from stromal cells by discontinuous PercollTM (GE Healthcare, cat. 17-0891-01) gradient centrifugation method.<br>Kidneys and spleen of 7-day-old pups were minced and digested with 50 µg/ml DNase 1 and 1 mg/ml Collagenase D in Hanks' solution at +37 °C for 45 min.<br>Blood from newborn and 7-day-old mice was collected by bleeding the body to heparin-PBS (50 µl of 100 IU/ml heparin in 500 µl of PBS). The postnatal blood was drawn by cardiac puncture into heparinized syringes and erythrocytes were lysed from the blood and spleen samples.<br>Postnatal testes were minced and digested with 50 µg/ml DNase 1 and 1 mg/ml Collagenase D in Hanks' solution at +37°C for 45 min. Kidneys were homogenized to RPMI-1640 medium in gentleMACS C-tube (Miltenyi Biotech) with gentleMACS Dissociator (Miltenyi Biotech) and leukocytes were isolated by OptiprepTM (Sigma-Aldrich, cat. D1556) gradient centrifugation. The bone marrow was isolated by gently crushing the femurs. Finally, the isolated cells were washed and suspended in PBS and all cell suspensions were filtered through a silk cloth (pore size 77 µm).
Instrument	The cells were analyzed with a BD LSRFortessa flow cytometer which was calibrated using compensation beads for each different antibody cocktail, or with HeliosTM, CyTOF system (Fluidigm).
Software	All flow cytometric data were analyzed using Flow Jo software (TreeStar). Mass cytometry data was either analyzed and visualized with Cytobank (https://www.cytobank.org) or with VorteX clustering environment (https://github.com/nolanlab/vortex/tag/29-Jun-2017; Published in Nat Methods. 2016 June ; 13(6): 493-496. doi:10.1038/nmeth.3863) and visualized with Gephi 0.9.1 (https://gephi.org).
Cell population abundance	The abundance of the cell populations is given as percentages.
Gating strategy	Cell were first defined by size and side scatter using FSC/SSC gating. Next, singlets were determined and dead cells were excluded using Live/Dead cell staining. Prenatal and newborn testis and brain macrophages were determined by gating on CD45+ and F4/80+. Postnatal testis macrophages were determined by gating on CD45+ _CD11b+_F4/80+_CD206+/-_MHC II+/-. Kidnay and spleen macrophages were determined by gating on CD45+ _CD11b+_F4/80Hi/int. Blood, bone marrow and testis monocytes were determined by gating on CD45+ _CD11b+_Ly6C+.<br>In mass cytometry all cells were first gated Cell ID positive, Cisplatin negative, newborn testis was gated CD45+_CD11b+ and F4/80+. Postnatal testis was gated as CD45+_CD11b+.

- [x] Tick this box to confirm that a figure exemplifying the gating strategy is provided in the Supplementary Information.
